# Antisickling Drugs Targeting βCys93 Reduce Iron Oxidation and Oxidative Changes in Sickle Cell Hemoglobin

**DOI:** 10.3389/fphys.2019.00931

**Published:** 2019-07-24

**Authors:** Tigist Kassa, Francine Wood, Michael Brad Strader, Abdu I. Alayash

**Affiliations:** Laboratory of Biochemistry and Vascular Biology, Center for Biologics Evaluation and Research, Food and Drug Administration, Silver Spring, MD, United States

**Keywords:** sickle cell anemia, hydroxyurea, L-glutamine, Endari, 4,4′-Di(1,2,3-triazolyl) disulfide (TD-3), 5-hydroxymethyl-2-furfural, cysteine 93, polymerization

## Abstract

Sickle cell disease is a genetic blood disorder caused by a single point mutation in the β globin gene where glutamic acid is replaced by valine at the sixth position of the β chain of hemoglobin (Hb). At low oxygen tension, the polymerization of deoxyHbS into fibers occurs in red blood cells (RBCs) leading to an impaired blood vessel transit. Sickle cell hemoglobin (HbS), when oxidized with hydrogen peroxide (H_2_O_2_), stays longer in a highly oxidizing ferryl (Fe^4+^) form causing irreversible oxidation of βCys93 to a destabilizing cysteic acid. We have previously reported that an antisickling drug can be designed to bind specifically to βCys93 and effectively protect against its irreversible oxidation by H_2_O_2_. Here, we report oxygen dissociation, oxidation, and polymerization kinetic reactions for four antisickling drugs (under different preclinical/clinical developmental stages) that either site-specifically target βCys93 or other sites on the HbS molecule. Molecules that specifically bind to or modify βCys93, such as 4,4′-di(1,2,3-triazolyl) disulfide (TD-3) and hydroxyurea (HU) were contrasted with molecules that target other sites on Hb including 5-hydroxymethyl-2-furfural (5-HMF) and L-glutamine. All reagents induced a left shift in the oxygen dissociation curve (ODC) except L-glutamine. In the presence of H_2_O_2_ (2.5:1, H_2_O_2_:heme), both TD-3 and HU reduced the ferryl heme by 22 and 37%, respectively, which corresponded to a 3- to 2-fold reduction in the levels of βCys93 oxidation as verified by mass spectrometry. Increases in the delay times prior to polymerization of HbS under hypoxia were in the following order: TD-3 > HU > 5-HMF = L-glutamine. Designing antisickling agents that can specifically target βCys93 may provide a dual antioxidant and antisickling therapeutic benefits in treating this disease.

## Introduction

Single amino acid replacement (glutamic acid → valine) in sickle cell disease (SCD) causes substantial reduction in the solubility of deoxyHbS leading to polymerization of tetramers into long fibers that decrease RBC membrane deformability. Distortion of the RBC into a rigid “sickle”-shaped cell leads to obstruction of blood flow in the microvasculature. Sickling and unsickling episodes cause damage to the cell membrane, decreasing the elasticity of the cell and its ability to return to a normal biconcave disc shape when normoxic conditions are restored (unsickling) (Rees et al., 2010).

Several antisickling strategies have been proposed to avoid or to minimize sickling of RBCs by directly targeting the HbS polymerization process. This includes blocking intermolecular contacts in the fiber, inducing the synthesis of non-polymerizing fetal Hb (HbF), shifting the allosteric transition in favor of non-polymerizing HbS oxyforms, and reducing 2,3-Diphosphoglycerate and HbS concentrations (Eaton and Bunn, 2017).

HbS polymerization is believed to contribute to the molecular pathogenesis associated with acute and chronic manifestations of SCD. It is not surprising that most efforts directed toward finding a treatment have focused on the design of small antisickling molecular agents that can permeate RBCs and directly inhibit polymerization (Eaton and Bunn, 2017). It has been recognized for some time that the oxidative milieu within RBCs (in SCD patients) can be a source of toxic reactive oxygen pieces (ROS) (Mahaseth et al., 2005). Among these recently identified internal sources for ROS are NADPH oxidase subunits and mitochondria that are retained by mature SS RBCs (George et al., 2013; Jagadeeswaran et al., 2017). This extra oxidative burden within SS RBCs leads to Hb structural instability, accelerated autoxidation, and heme loss (Hebbel et al., 1988; Kassa et al., 2015). Hb oxidation side reactions within SS RBCs and RBC-derived microparticles (MPs) and the release of free Hb/heme further contribute to the complex disease pathophysiology (Alayash, 2018; Jana et al., 2018). Specifically, Hb is able to participate (inside and outside RBCs) in pseudoenzymatic radical side reactions which include the formation of a persistent and highly oxidizing ferryl state (HbFe^4+^) as part of this pseudoperoxidative cycle. In addition to targeting its own β subunits (specifically the hotspot βCys93), ferrylHb and its associated radical (HbFe^4+^) actively interact with other biological molecules (Kassa et al., 2015).

We have recently reported a newly developed reagent (di(5-(2,3-dihydro-1,4-benzodioxin-2-yl)-4H-1,2,4-triazol-3-yl)disulfide) (TD-1) that exhibits a dual antisickling and antioxidant function by shielding βCys93 (the end point of radical formation in HbS) in addition to its allosteric modification of HbS (Nakagawa et al., 2014; Kassa et al., 2017). In this study, we investigated the antisickling and antioxidant properties of several antisickling drugs that are either clinically approved for use in the USA (hydroxyurea, and L-glutamine) or currently undergoing clinical and preclinical evaluation [5HMF, and the triazol disulfide (TD-3) (an analogue for TD-1) (Abdulmalik et al., 2005; Nakagawa et al., 2018)].

The use of hydroxyurea (HU) (**1**), in the treatment of SCD, was approved by the Food and Drug Administration in 1998. HU was found to increase the amount of HbF in patient blood. However, the cellular mechanism(s) by which this occurs are not completely understood, and other mechanisms may also account for the clinical benefits attributed to this agent (Ferrone, 2016). The increase in the amount of non-polymerizing HbF dilutes out HbS in patient’s own RBCs that result in a decrease in the degree of polymerization and consequently relieving some of the symptoms associated with the disease (Eaton and Bunn, 2017). Another possible protection mechanism that HU may provide is through the S-nitrosylation of βCys93, which we have recently reported (Jana et al., 2018).

Endari, an oral L-glutamine therapy (**2**), is the another FDA approved drug intended to reduce acute complications of SCD in adults and children older than 5 years (Riley et al., 2018). Oral L-glutamine therapy has been suggested to benefit SCD patients as it potentially increases the activity of NAD synthesis, thus countering the oxidant-dependent pathophysiology of sickled RBCs. Redox potential of RBCs is defined by the ratio of NADH to the sum of NADH plus NAD+. This change in NAD redox potential decreases RBCs sensitivity to oxidative stress. This increased rate of NAD synthesis can therefore compensate for increased oxidative stress (Niihara et al., 1997).

5-Hydroxymethylfurfural (5-HMF) (**3**) is a naturally occurring aromatic aldehyde that has been reported to bind to the Hb molecule producing a left shift in the oxygen equilibrium curve, which reduces erythrocyte sickling in animal models (Abdulmalik et al., 2005). 5HMF binds reversibly *via* a Schiff-base to the N-terminal Val 1 of Hb alpha chains and thus allosterically modulates oxygen affinity. This agent is currently under clinical evaluation (Abdulmalik et al., 2005).

The triazole disulfide compound 4,4′-di(1,2,3-triazolyl) disulfide (TD-3) (**4**), which has higher aqueous solubility than TD-1 (while maintaining the ability to increase Hb oxygen affinity), is currently under preclinical investigation (Nakagawa et al., 2018). The crystal structures of carboxy- and deoxy-forms of human adult Hb (HbA), each complexed with TD-3, reveal that one molecule of the TD-3 monomeric thiol form forms a disulfide bond with βCys93, which inhibits the salt-bridge formation between β-Asp94 and β-His146. This inhibition of salt bridge formation stabilizes the R-state and destabilizes the T-state of Hb (Nakagawa et al., 2018).

Here, we compared these four antisickling agents and found that compounds that directly or indirectly interact with βCys93 prevent irreversible oxidation of cysteine to cysteic acid and provide additional protection against oxidants. It is feasible that one can therefore design new drugs targeting several processes including sickling and oxidative pathways as part of a new therapeutic modality to treat SCD.

## Materials and Methods

### Materials

All chemicals and reagents were purchased from Sigma-Aldrich (Saint Louis, Missouri) or from Fisher Scientific (Pittsburgh, Pennsylvania) unless otherwise specified. Blood samples used in this study were obtained with consent from patients homozygous for HbS (off and on hydroxyurea treatment) during routine clinic visits at the National Institutes of Health (NIH), and blood from healthy donors (homozygous for HbA) was also obtained from the NIH Blood Center. Hb was purified as previously described using anion and cation chromatography (Meng and Alayash, 2017). Purified sickle Hb was also obtained from Sigma-Aldrich. AFSC control containing mix of HbA, HbF, HbS, and HbC was purchased from Analytical Control Systems, Inc.

### Experimental Procedures

#### Measurement of Oxygen Dissociation Curves

The oxygen dissociation curves (ODC) of suspensions of SS/AA cells were determined with a Hemox TM Analyzer (TCS Scientific Corp.) as previously described (Kassa et al., 2017). To determine the ODC of SS cells treated with the antisickling agents, cells were washed, packed, and re-suspended in plasma to a hematocrit of approximately 20%. The suspensions were incubated with 0 or 2 mmol/L antisickling agents at 37°C for 15 min (for TD-3) or 1 h (for the rest of the agents). Approximately 120 μl of each suspension was added to 3 ml of Hemox buffer, pH 7.4, in a cuvette and subjected to ODC analysis at 37°C. A 120 μl of the 20% AA or SS RBCs suspension with 3 ml Hemox buffer was used as a control. Analysis was performed using triplicate samples.

#### Measurement of HbS Polymerization Kinetics

The polymerization assay was carried out using commercially available HbS. The HbS solution was treated with the antisickling agent prior to addition to the deoxygenated buffer solution as previously reported (Kassa et al., 2017). Two equivalence (4.8 mM) of TD-3 and four equivalence of the 5HMF, HU, and glutamine (9.6 mM) were used for the treatment of HbS (2.4 mM) solutions. Temperature of the treated stock HbS solutions were then dropped to 0°C by keeping the solution in an ice bucket. These reactions were kept at high concentration of phosphate buffer (1.8 M, pH 7.3) in a 1 ml volume sealed cuvette with nitrogen gas bubbled for 60 min to deoxygenate the solution. A stock Hb solution was then aliquoted (at 0°C) in the cuvettes through a rubber septum with a Hamilton syringe to make final concentration of Hb in the cuvette as 120 μM. The cuvette was placed in a preheated sample holder in the spectrophotometer (30°C). The change in turbidity was measured with a temperature-controlled photodiode array spectrophotometer at 700 nm. The optical density at 700 nm was plotted against time to give a sigmoidal curve that shows the delay time of polymerization.

#### Hydrogen Peroxide-Mediated Oxidation of HbS

The effects of antisickling agents on HbS oxidative stability were examined using a temperature-controlled photodiode array spectrophotometer (Agilent 8453, Santa Clara, California, US). H_2_O_2_-induced oxidation of ferrous Hb was monitored with and without prior treatment of HbS with the four antisickling agents. Determination of the H_2_O_2_-induced ferryl Hb species formation was done following our previously described method (Kassa et al., 2017). Pre-incubated HbS solution (60 μM) with a given antisickling agent (60 μM) at 37°C for the appropriate time was then brought to room temperature and subsequently treated with increasing concentrations of H_2_O_2_ (0, 60, 150, 300, or 600 μM) followed by incubation for 5 min at 25°C. Catalase (200 units/ml) was added to the solution to terminate further oxidation reactions and remove excess H_2_O_2_. Absorbance spectra between 350 and 700 nm were measured to monitor the reaction. To determine the amount of ferryl intermediate produced, 2 mM sodium sulfide (Na_2_S) was added immediately to derivatize the ferryl heme into sulfHb that can be monitored by the appearance of absorption maxima at 620 nm. The concentration of sulfHb was calculated using an extinction coefficient of *ε* (620 nm) = 24.0 mM^−1^ cm^−1^ as previously reported (Berzofsky et al., 1971).

#### RP-HPLC Analysis of Lysates

Hb solutions were prepared by lysing packed RBCs with twice their volume of water. The solution was then centrifuged at 13,000 rpm for 5 min at 4°C. RP-HPLC analyses were performed with a Zorbax 300 SB C3 column (4.6 mm × 250 mm) using a Waters HPLC system consisting of a Waters 626 pumps, 2,487 dual-wavelength detector, and 600-s controller installed with Empower 2 software (Waters Corp, Milford, MA). The supernatant, containing Hb (20 μg) in 25 μl of water was loaded on the C3 column equilibrated with 35% acetonitrile containing 0.1% TFA (Buffer B). Globin chains were eluted with a gradient of 35–53% ACN within 80 min at a flow rate of 1 ml/min. The eluent was monitored at 280 nm for globin chains and at 405 nm for the heme components.

#### Quantitative Mass Spectrometric Analysis of βC93 Oxidation in HbS

Quantitative mass spectrometry (MS) analysis was performed utilizing 60 μM (heme) HbS. To study the effect of all four antisickling agents on H_2_O_2_ mediated βCys93 amino acid oxidation, the following experimental conditions were utilized for all MS experiments described below. Experiment 1, controls: HbS was incubated in air equilibrated PBS buffer without H_2_O_2_. Experiments 2 and 3: HbS was incubated with 2.5 and 10 molar excess of H_2_O_2_ per heme. Experiments 4 and 5: HbS was incubated with each antisickling agent with 2.5 and 10 molar excess of H_2_O_2_ per heme. All oxidation reactions were carried out in phosphate buffer saline (PBS), pH 7.4 at ambient temperature for 30 min. One microliter of 1 unit/μl catalase was added to remove excess H_2_O_2_ to quench oxidation from samples listed in experiments 2–5.

#### LC-MS/MS Analysis

All HbS samples (listed above in experiments 1–5) were digested with trypsin, desalted, and analyzed by mass spectrometry as previously described (Strader et al., 2014). Briefly, tryptic peptides were analyzed by reverse phase liquid chromatography mass spectrometry (RP LC/MS/MS) using an Easy nLC II Proxeon nanoflow HPLC system coupled online to a Q-Exactive Orbitrap mass spectrometer (Thermo Scientific). Data were acquired using a top10 method (for 60 min), dynamically choosing the most abundant precursors (scanned at 400–2,000 m/z) from the survey scans for HCD fragmentation.

Mass spectrometry was also used to confirm that HbS control samples were catalase free by searching data against the Swiss-Prot Human database (release 2014_03, containing 542,782 sequence entries) supplemented with the porcine trypsin sequence using the Mascot (version 2.4) search engine (Matrix Sciences, London, UK) as described previously (Strader et al., 2014). Variable modifications including cysteine trioxidation (+48 Da), methionine oxidation (+16), and S-nitrosylation (+29 Da; for hydroxyurea samples) were included for identifying oxidative modifications. Mascot output files were analyzed using the software Scaffold 4.2.0 (Proteome Software Inc.). Hb peptide identifications were accepted if they could be established at greater than 99.9% probability and contained at least two identified peptides. Probabilities were assigned by the Protein Prophet algorithm (Keller et al., 2002).

#### Intact Mass Analysis

Subunits of intact HbS with either 5-HMF or TD-3 adducts were isolated using RP-HPLC prior to LC-MS analysis. Samples were analyzed using a Q-Exactive as described above with the instrument configured to acquire data in full-MS mode with a resolution of 140,000 full width half maximum (FWHM). Isotopically resolved charge state envelopes (multiply charged ions of intact monomers) representing mass spectra of intact Hb subunits were deconvoluted using the protein deconvolution software Xtract (Thermo Scientific). The deconvoluted mass spectrum yielded a measured molecular mass that was within 0.025 Da (3 ppm) of the calculated value.

#### Quantitative MS Analysis

Peptides from LC-MS/MS data were analyzed to quantify changes in Hb under oxidative conditions as previously described (Strader et al., 2014). Each peptide was further validated by retention time reproducibility. All quantitative experiments were performed in triplicate, and standard deviations were obtained by averaging relative abundance data from three different experiments. Extracted ion chromatograms (XICs) were generated from the most abundant monoisotopic peak of isotopic profiles representing charged states of each peptide (oxidized and unoxidized). To construct XICs, Xcalibur (version 2.4) software was used with a designated mass tolerance of 0.01 Da, and mass precision set to three decimals. For relative quantification, the ratio of each isoform was calculated based on the sum of the XIC peak area from all forms, which was normalized to 100% and included all charge states and versions that result from different cleavage sites.

## Results

### Measurements of Oxygen Dissociation Curves

**Figure 1:**
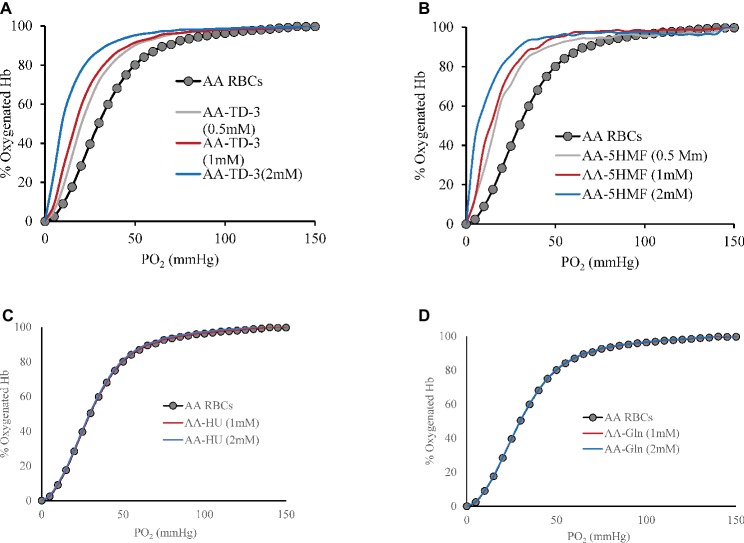
The effect of antisickling agent on the ODCs of AA intact cells. Suspensions of AA cell hematocrit (20%) were incubated in the presence of various concentrations of antisickling agent at 37°C for 15 min (TD-3) or 1 h (the other antisickling agents) prior to determination of the ODC using Hemox Analyzer. Oxygen dissociation curves (ODC) of AA cells with increasing molar ratio of TD-3, 5HMF, HU, and glutamine to Hb in heme are shown in **(A)**, **(B)**, **(C)**, and **(D)**, respectively.

**Figure 2:**
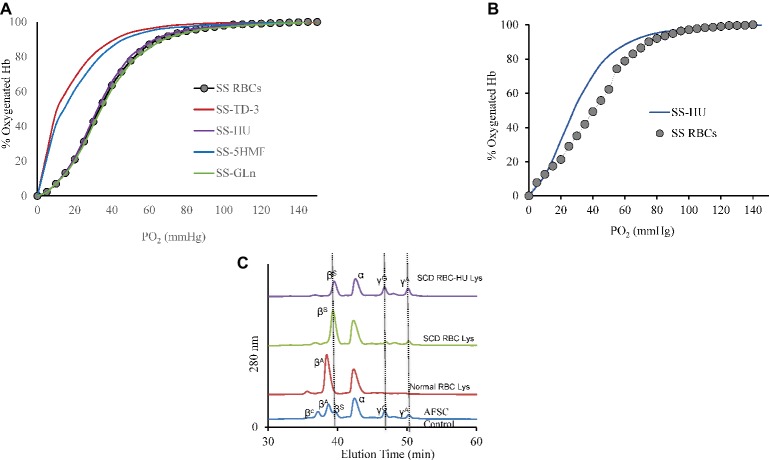
Oxygen dissociation curves (ODC) of SS intact cells after treatment with the antisickling agents. **(A)** Suspensions of SS cells [hematocrit (20%)] were incubated in the presence of antisickling agent at 37°C for 15 min (TD-3) or 1 h (the other antisickling agents) prior to determination of the ODC using Hemox Analyzer. Oxygen dissociation curves (ODC) of SS cells with 2 mM TD-3, 5HMF, HU, or glutamine is shown in the Figure. **(B)** Left-shift of the oxygen dissociation curve (ODC) of sickle cell patient blood who was on HU compared to patient who was not on treatment of HU. **(C)** RP-HPLC data of lysate from patient with and without HU treatment. Hb (0.96 mg) was injected into C3 column. The mobile phase consisted of (1) 0.1% TFA in water and (2) 0.1% TFA in acetonitrile. A gradient was programmed to increase from 35 to 53% Buffer B over 80 min. Solvents were mixed and run at a rate of 1 ml/min and absorbance was monitored at 280 and 405 nm. AFSC is monitored at 280 and is shown as control.

Effects of the four antisickling agents on the oxygen affinity of normal AA and SS RBCs were studied. Incubation of normal AA cells (20% Hct) with increasing concentrations of TD-3 (0, 0.5, 1, and 2 mM) shifted the ODC to the left in a dose-dependent manner (Figure 1A). Treatment of the RBCs with the highest TD-3 dose (2 mM) resulted in a *P*_50_ reduction from 28.4 to 9.8 mmHg. Similar reduction in *P*_50_ values was observed in the ODCs of SS cells that had been pre-incubated with TD-3 (2 mM) (Figure 2A); the *P*_50_ value of the SS cells was reduced from 34.2 to 9.6 mmHg. These results demonstrated the ability of TD-3 to permeate through RBC membranes and to interact with intracellular HbS, consistent with early reports (Nakagawa et al., 2018). In the absence of TD-3 (control), the ODC of Hb solutions (data not shown) and RBCs showed a typical sigmoid curve; however, pre-incubation of cells with TD-3 induced a change in shape of the curve from sigmoid to hyperbolic with increasing TD-3 concentrations, indicating a loss in cooperativity as reflected by reduced values of the Hill coefficients (as shown in Table 1).

**Table 1:** Oxygen equilibrium data for RBCs treated with antisickling agents.

Antisickling agent (2 Mm)	AA cells (25% Hct) *P*_50_ (mmHg)	AA cells Δ*P*_50_ (mmHg)	AA cells Hill coefficient (*n*)	SS cells (25% Hct) *P*_50_ (mmHg)	SS cells Δ*P*_50_ (mmHg)	SS cells Hill coefficient (*n*)
Control (0)	28.4 ± 0.19	–	2.81 ± 0.07	34.2 ± 0.71	–	2.86 ± 0.02
TD-3	9.8 ± 0.30	18.6	1.66 ± 0.01	9.6 ± 0.40	24.6	1.67 ± 0.03
5HMF	8.7 ± 0.21	19.7	1.25 ± 0.04	10.8 ± 1.26	24.1	1.39 ± 0.05
HU	28.1 ± 0.02	0.3	2.86 ± 0.03	33.97 ± 0.57	0.23	2.86 ± 0.04
Gln	28.1 ± 0.08	0.3	2.84 ± 0.04	33.73 ± 0.47	0.47	2.86 ± 0.04

The effect of 5HMF on oxygen affinity of intact RBCs was also studied under the same experimental conditions described above. There was a reduction in the *P*_50_ from 28.4 to 8.7 mmHg when AA cells were treated with 5HMF (Figure 1B). Similarly, a reduction in the *P*_50_ value derived from ODCs of SS cells pre-incubated with 5HMF (2 mM) was observed (Figure 2A); the *P*_50_ value of the SS cells was reduced from 34.2 to 10.8 mmHg. We observed changes not only in the position but also in the shape of ODC with increasing concentrations of 5HMF.

*In vitro* incubation of AA or SS RBCs (20% Hct) with HU or L-glutamine (2 mM) did not induce a shift (left or right) in the ODCs as shown in Figures 1C,D. Similarly, no effects on the ODCs were observed with purified Hb (data not shown). To demonstrate the long-term effects on HbF induction by HU *in vivo*, we measured the ODC of HU-treated patient blood samples which showed a clear left shift in the ODC compared to a sample from SS patient who was not on HU treatment (Figure 2B). The *P*_50_ value had decreased from 40.95 to 32.51 mmHg, respectively. This reduction in *P*_50_ value and the left shift is attributed to the presence of HbF in the patient’s blood. This result was further confirmed by RP-HPLC analysis. For these experiments, the HU-treated patient lysate revealed the presence of HbF γ subunits (~20% HbF). Under our chromatographic conditions, the heme component for all samples had an elution time of 15 min whereas β and α chains of HbA eluted at 38 and 44 min, respectively, and the β and α chains of HbS eluted at 40 and 44 min, respectively. The β^C^, γ^G^, and γ^A^ eluted at 36, 48, and 50 min, respectively (Figure 2C). However, the profile of lysates from the patient who was not on HU treatment did not show much of the γ subunit peak. *P*_50_ and Hill coefficient values for HU or glutamine-treated RBCs are listed in Table 1.

### Effects of Antisickling Reagents on the Polymerization Kinetics of HbS

**Figure 3:**
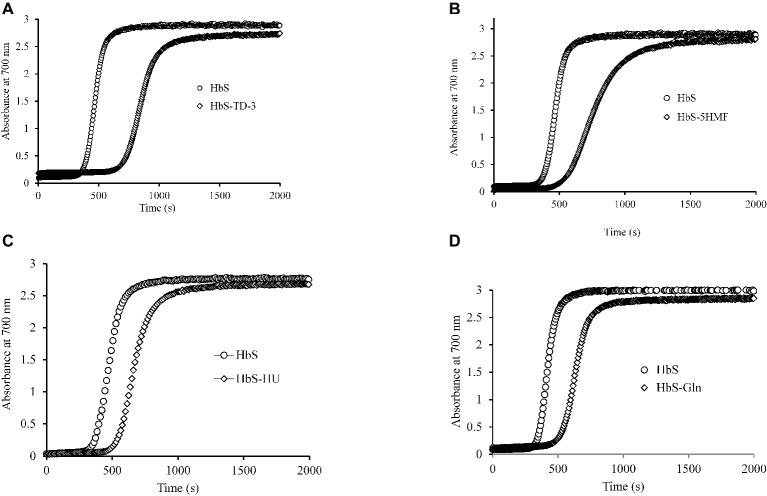
Effects of antisickling agents on the kinetics of deoxyHbS polymer formation. Solutions of deoxyHbS 120 μM were rapidly heated from 0 to 30°C. Polymer formation was assessed by light scattering at 700 nm in 1.8 M phosphate buffer at pH 7.3. **(A)** Polymerization of deoxyHbS solution in the presence and absence of TD-3. **(B)** In the presence of 5HMF. **(C)** In the presence of hydroxyurea. **(D)** In the presence of glutamine.

HbS polymerization begins with a prominent latency period, called a “delay time” followed by abrupt polymer formation (Eaton and Bunn, 2017). Previous studies have shown that an increase in the concentration dependent delay time for HbS polymer formation has a therapeutic effect (Mozzarelli et al., 1987) by virtue of preventing most cells from sickling *in vivo*. We tested the polymerization kinetics of treated and untreated oxyHbS with all four antisickling agents. Delay time results for treated samples were compared with non-treated HbS samples and are presented in Figure 3. Our results indicate that all four antisickling agents increased the delay time when compared with control HbS. The delay time plot (absorbance changes at 700 nm as a function of time) for HbS treated with TD-3 was shifted to the right of the HbS control (Figure 3A). The delay time for the untreated and TD-3 treated HbS was 315 and 630 s, respectively. These results suggest that TD-3 increased the delay time by 315 s indicating that TD-3 treated HbS polymerizes slower than the untreated HbS (control). The same trend was observed with the other antisickling agents. HU increased the delay time by 170 s while both 5HMF and glutamine increased the delay time by 158 s. Taken together, these data confirm that these antisickling agents inhibit *in vitro* HbS polymerization and suggest that they may also delay *in vivo* HbS polymerization.

### Hydrogen Peroxide-Induced Oxidation of HbS

**Figure 4:**
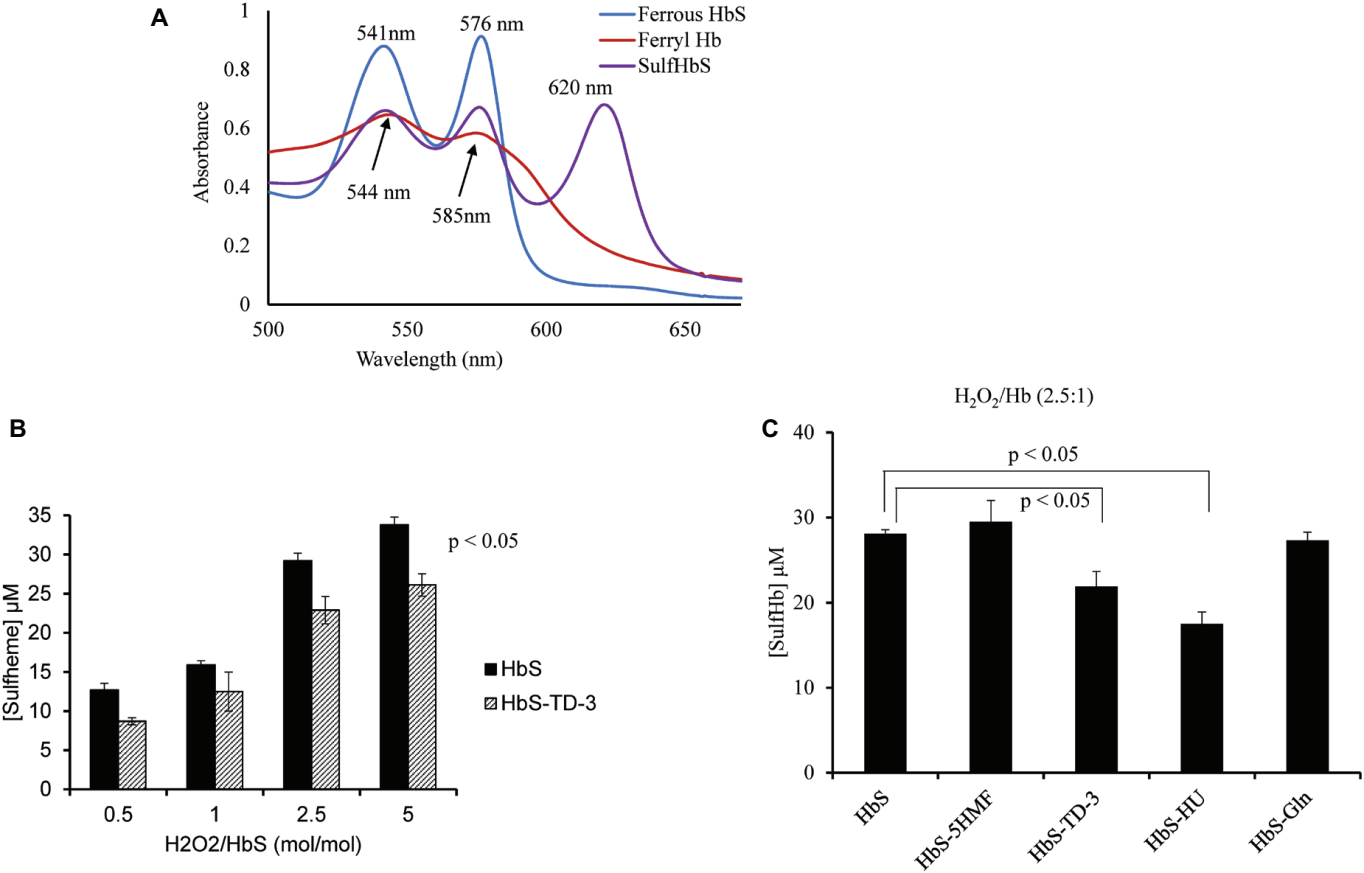
Spectral analysis of TD-3 effect on ferryl hemoglobin formation. **(A)** Absorbance spectra obtained upon treatment of HbS (60 μM) (blue line) with 150 μM H_2_O_2_. Ferryl Hb spectrum (red) is characterized by new emerging peaks at 544 and 585 nm, respectively, and a flattened region between 600 and 700 nm. Absorbance spectra obtained immediately after addition of Na_2_S (2 mM) to convert HbS to sulfHb (purple) that exhibits a characteristic strong peak at 620 nm. **(B)** Levels of sulfHb are plotted in a bar graph; non-treated HbS (black) and TD-3 treated (pattern). **(C)** Sulfheme levels produced from HbS treated with the antisickling agents.

In the presence of H_2_O_2_, Hb is transformed into the higher oxidation ferryl Hb (Fe^4+^) form and the globin associated radical. Both the ferryl Hb (Fe^4+^) form and the globin-associated radical are strong oxidants that can induce irreversible oxidation of “hotspot” amino acids near the heme region with βCys93 being the most impacted. The irreversible oxidation of βCys93 to cysteic acid in the presence of H_2_O_2_ destabilizes Hb (Kassa et al., 2015). In this study, we estimated ferryl heme levels that were formed with H_2_O_2_ treatment in the presence and absence of each antisickling agent. Sodium sulfide was used to derivatize the transient ferryl Hb to a stable sulfHb intermediate that could then be spectrophotometrically measured. A typical spectral transition of the reaction of ferrous HbS (60 μM) with H_2_O_2_ (150 μM) representing ferryl formation and its conversion to sulfHb is shown in Figure 4A. Figure 4B shows sulfHb levels in HbS solutions treated with TD-3 in the presence of increasing concentrations of H_2_O_2_. The levels of sulfHb produced from TD-3-treated HbS are lower than that of the non-treated HbS at all concentrations of H_2_O_2_ (*p* ≤ 0.5). Similar trends were seen in the HU-treated samples in response to increasing H_2_O_2_ concentrations. Figure 4C compares the effects of all antisickling agents on the amount of ferryl produced with the reaction of H_2_O_2_ (at a ratio of H_2_O_2_:Hb of 2.5:1). The results summarized in Table 2 indicate that HbS treatment with TD-3 and HU reduced ferryl levels by 22 and 37%, respectively, confirming the antioxidant property of these antisickling agents. 5HMF and L-glutamine, on the other hand, showed no effects on ferryl Hb levels produced by the treatment with H_2_O_2_.

**Table 2:** SulfHb produced by the reaction of hydrogen peroxide (μM) with sickle Hb in the presence and absence of antisickling agent.

H_2_O_2_/Hb	HbS	HbS-TD-3 (% Reduction)	HbS-5HMF	HbS-HU (% Reduction)	HbS-Gln
0.5	12.7 ± 0.55	8.7 ± 0.95 (31.5%)	13.2 ± 1.45	8.0 ± 1.41 (37.0%)	12.5 ± 0.51
1	23.9 ± 0.45	12.5 ± 0.96 (47.7%)	24.2 ± 1.82	10.4 ± 1.05 (56.5%)	25.3 ± 0.85
2.5	28.1 ± 0.45	21.9 ± 1.74 (22.1%)	29.5 ± 2.50	17.5 ± 1.42 (37.7%)	27.3 ± 0.80
5	38.9 ± 0.44	26.1 ± 1.45 (32.9%)	36.4 ± 2.15	23.9 ± 1.22 (38.6%)	39.0 ± 0.92

### Mass Spectrometric Analysis to Evaluate Antioxidant Properties of Anti-Sickling Agents

**Figure 5:**
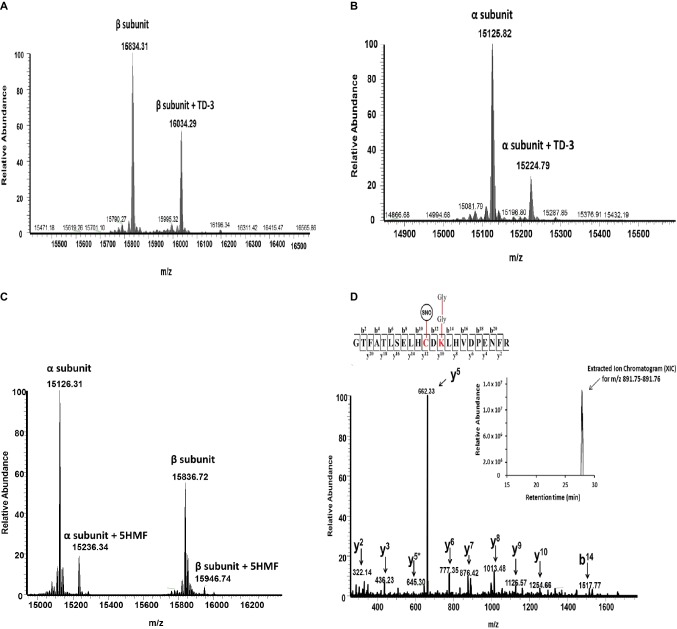
Representative mass spectrometry data confirming the modification of HbS by TD-3, 5HMF, and HU. Intact mass (top-down) and LC/MS/MS (bottom-up) analysis were utilized to elucidate how the antisickling reagents interact with HbS. (**A** and **B**) Intact mass data confirm that TD-3 substantially modifies both β and α subunits. **(C)** Intact mass data confirms that 5HMF modifies both α and β subunits. **(D)** MS/MS fragmentation spectrum representing the y and b ions matched to the modified tryptic peptide GTFATSELHCDKLHVDPENFR (residues 83–104) from HbS incubated with HU. This peptide contains the diglycine signature modification (at βK96) associated with ubiquitination and S-nitrosylation (at βC93). The inset represents the extracted ion chromatogram representing the S-nitrosylated peptide.

Using mass spectrometric analysis, we first verified the binding of these antisickling agents to HbS. Figure 5 shows that TD-3, 5HMF, and HU modified HbS. For example, Panel A and B show intact mass (top-down) data confirming that TD-3 interacted with the β subunit and α subunit, respectively. These results support previous studies that linked the TD-3 modification specificity to βCys93 (Nakagawa et al., 2018); this report is the first however to show that TD-3 also modifies the α subunit. Similar βCys93 oxidation levels from this study at both 2.5X and 10X H_2_O_2_ conditions (see below) also correlate with the intact mass data indicating that the TD-3 β subunit modification stoichiometry was likely similar for both conditions.

Figure 5C also confirms previous reports that 5HMF reacts specifically with α N-termini (Abdulmalik et al., 2005). Our data are the first to also show that 5HMF also forms a covalent bond with the β N-terminus. Finally, Figure 5D shows the LC/MS/MS fragmentation spectrum (bottom-up) and corresponding inset representing the extracted ion chromatogram of the S-nitrosylated peptide (i.e., addition of 29 Da) supporting our earlier studies which link HU treatment to βC93 specific S-nitrosylation (Jana et al., 2018). It has previously been established that the surface amino acid βCys93 is an endpoint for free radical induced Hb oxidation and consequently incurs prevalent H_2_O_2_ induced oxidative changes (Jia et al., 2007; Pimenova et al., 2010; Strader and Alayash, 2017). Additionally, we have previously shown, *via* LC/MS/MS analysis, that HbS oxidative toxicity (compared to HbA) is linked to irreversible βC93 oxidation to cysteic acid at the amino acid side chain (Kassa et al., 2015). Next, LC/MS/MS quantification of βCys93 oxidation was therefore utilized (as an “oxidation reporter”) to evaluate the potential antioxidant properties of each antisickling agent.

**Figure 6:**
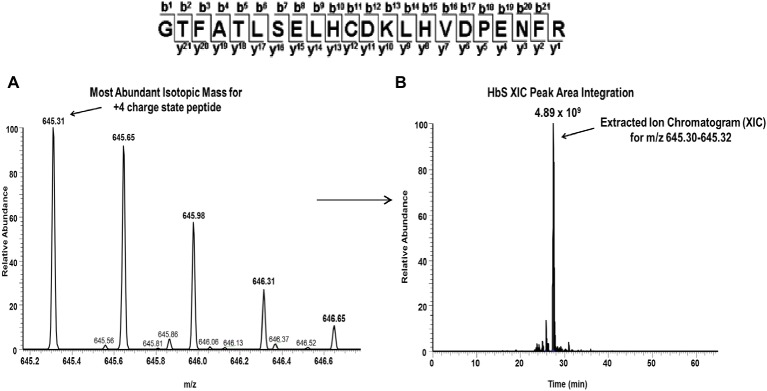
Representative extracted ion chromatogram (XIC) data of the oxidized Cys93 tryptic peptide HbS (residues 83–104) after treatment with peroxide. For quantitative mass spectrometry experiments, the charge state isotopic profile of all βC93 peptides (and their different charge states listed in Table 5) was analyzed as shown in this figure to quantify changes under different oxidative conditions. The panels in this figure represent data after experimental treatment with 10X H_2_O_2_. **(A)** Isotopic profile for the Cys93 containing +4 charge state peptides (645.31 m/z) listed above (residues 83–104). **(B)** Representative XIC and peak area integration for the oxidized HbS Cys93 peptide generated from the ion current of the most abundant monoisotopic peak shown in panel A.

For each βCys93 containing peptide charge state identified by Mascot database searches (see Table 3), extracted ion chromatograms (XICs) were generated from the most abundant monoisotopic peak of each peptide isotopic profile, and the resulting ratio differences were compared. For example, the most abundant mono-isotopic peak (645.31 m/z) in Figure 6A for the oxidized +4 βCys93 peptide, GTFATLSELHCDKLHVDPENFR, (treated with 10X H_2_O_2_) was used to construct the XIC shown in Figure 6B. Because βCys93 exists in either the oxidized or unoxidized form after treatment with 10X H_2_O_2_, the relative abundance of both isoforms was calculated based on the sum of the XIC peak area from all charged isoforms of βCys93 peptides. For each experimental condition analyzed by LC/MS/MS, XICs were generated in a similar manner (for all βC93 peptide charge states listed in Table 3), and the XIC peak area integration values were determined for relative quantification as previously described (Kassa et al., 2015; Strader and Alayash, 2017; Meng et al., 2019). Table 4 represents the calculated ratio values for these studies based on the sum of XIC numerically integrated peak area of all βCys93 peptide charge states (oxidized and unmodified) to be 100%. As shown in this table, H_2_O_2_ addition (2.5- and 10-fold in excess) led to increased βC93 oxidation; the level of oxidation in the controls was similar to our previous HbS study (Kassa et al., 2015). With the exception of glutamine and 5HMF, the other two agents (TD-3 and HU) reduced βC93 oxidation by providing anti-oxidant protection; TD-3 modification resulted in the most substantial decrease in βC93 oxidation (by ~3-fold) followed by HU which reduced βC93 oxidation by ~2-fold.

**Table 3:** C93 peptides including different charge states.

Peptides	Modified residue	(+) Charge state	m/z
^83^GTFATLSELHCDK^96^	βCys93	2	735.33
^83^GTFATLSELHCDKLHVDPENFR^104^	βCys93	3	860.06
		4	645.31
		5	518.25

**Table 4:** Hydrogen peroxide mediated oxidation of HbS βC93.

Antisickling agent + HbS	C93 ox 2.5X H_2_O_2_	C93 ox 10X H_2_O_2_
HbS control	36.4 ± 1.4%	69.1 ± 7.3%
5HMF	33.1 ± 2.7%	61.0 ± 0.4%
TD-3^*^	11.8 ± 1.8%	12.5 ± 0.4%
Hydroxyurea (HU)	17.2 ± 0.9%	32.1 ± 1.7%
Glutamine (Gln)	35.0 ± 0.1%	67.9 ± 1.2%

* *TD-3 modifies Cys93; these values represent oxidation from unlabeled Cys93*.

## Discussion

The sickling/unsickling cycles in the microvasculature result in greater (almost 2-fold) amounts of ROS (mainly O_2_^−^, and H_2_O_2_) that accumulate within SS RBCs (Jana et al., 2018). Consequently, RBC antioxidative enzyme activities (primarily SOD and catalase) increase in response to ROS-induced oxidative stress. Increased expression of NADPH oxidase-derived ROS (George et al., 2013) and the retention of active mitochondria in mature RBCs may also cause direct oxidative damage to a variety of subcellular structures which ultimately leads to increased RBC fragility and hemolysis (Jagadeeswaran et al., 2017). We have recently shown that changes in antioxidative enzymes and consequent posttranslational changes in the Hb molecule extend into the RBC-derived MPs that are circulating in sickle cell blood (Jana et al., 2018). These Hb-dependent oxidation reactions were also found to drive cytosolic internal and RBC membrane changes including alternation in band 3 activities.

Despite our growing mechanistic understanding of HbS polymerization and the resulting effects on RBCs in circulation, current treatment options do not fully address the complex molecular manifestations of SCD. It was recently suggested, however, that systems-oriented multi-agent strategies should be developed to counter the multifaceted aspects of this disease by designing new drugs targeting several of these processes. This includes agents that reactivate HbF, anti-sickling agents, anti-adhesion agents, modulators of ischemia-reperfusion (and oxidative stress), agents that counteract free Hb and heme, anti-inflammatory agents, anti-thrombotic agents, and anti-platelet agents (Telen et al., 2019).

The oxidative stress is partly driven by the formation of highly oxidizing ferryl heme and its associated protein cation radicals in parent RBCs and within RBC-derived MPs (Jana et al., 2018). These internal reactions result in irreversible amino acid oxidation of the protein in the area known as “oxidative hotspots” (specifically the βCys93 side chain) that contribute to the partial collapse of β subunits, unfolding and degradation of Hb, and the ultimate release of heme. The amino acid βCys93 occupies an important and crucial position at the β/α interface and is critically involved in the Hb R to T transition. βCys93 has been shown to be extensively and irreversibly oxidized to cysteic acid in the presence of relatively low levels of H_2_O_2_. Moreover, βCys93 is more amenable to oxidative attack by ferryl Hb and its protein radicals (Jia et al., 2007).

Since βCys93 plays a key role in Hb allosteric, antisickling, and oxidative modulation (Jia et al., 2007; Omar et al., 2015), we tested whether currently approved drugs and other compounds that are under development may provide combined antisickling and antioxidative functions by targeting βCys93. Toward this goal, we contrasted two sets of compounds that are known to site specifically bind/modify βCys93 (i.e., HU and TD3) with those that non-site specifically interacts with the protein (i.e., 5HMF and L-glutamine).

The therapeutic benefits of HU have been attributed to its ability to increase HbF levels in patient blood. It has also been reported that HU exhibits antioxidant properties by protecting patient RBCs against oxidative stress (Agil and Sadrzadeh, 2000; Nader et al., 2018). In an experiment designed to investigate potential HU antioxidant characteristics, HU-treated Townes sickle cell mice (fed a diet containing HU for 10 days) showed a remarkable reduction in MP formation, band 3 modifications, and Hb posttranslational modifications (including reduction in βCys93 oxidation) in their hemolysates (Jana et al., 2018). In the presence of HU, a causative relationship between ferryl heme and the degree of cysteic acid formation were clearly established in our current *in vitro* studies consistent with these earlier observations (Jana et al., 2018). Previous *in vitro* and *in vivo* EPR studies have documented NO release from HU through nitrosyl Hb formation (Kim-Shapiro et al., 1999; Huang et al., 2002; King, 2004). We have also previously shown that NO released from an NO donor sodium 2-(N,N-diethylamino)-diazenolate-2-oxide (DEANO) binds specifically to βCys93 (Hrinczenko et al., 2000). The electrospray mass spectrometry data in this study also confirm the presence of S-nitrosylation (corresponding to a 29-Da increase consistent with NO binding) of both the β chains but not of the α chains. Hb S-nitrosylation at βCys93 has been shown to have stabilizing effects on Hb since it shields this amino acid from ferryl radical reactivity (Jana et al., 2018). This may provide an additional molecular interpretation for the reported efficacy of this drug in treatment of SCD (Ferrone, 2016). This newly antioxidative benefit (associated with use of HU) is independent of its well-known HbF induction as we have documented in HU-treated patient blood who was on HU therapy regimen. The most striking finding in this work, however, is the correlation between the HU induced reduction in both ferryl heme and βCys93 oxidation levels (with increasing H_2_O_2_ concentrations) when comparing the same experimental conditions with the HbS control.

The basis of the antisickling action of TD-3 was found to be due to a disulfide formation with βCys93 of HbS’s β subunit (Nakagawa et al., 2018). The crystal structure of HbACO complexed with TD-3 revealed that MT-3 (the monomer) forms a disulfide bond with the βCys93 side chain thiol. The nitrogen atoms of the triazole ring form a hydrogen-bond interaction and water-mediated hydrogen-bond interactions with protein oxygen atom in the region. Our mass spectrometric analysis of intact and digested peptides of HbS confirms that TD-3 substantially modifies both β and α subunits of HbS. While intact mass data indicated that the TD-3 modification stoichiometry was not 100%, TD-3 provided the greatest degree of anti-oxidant protection as evidence by a sustained reduction in ferryl heme and its target β subunit Cys93. The labeling and protection of βC93 was further substantiated by the fact that βC93 oxidation was substantially reduced in TD-3 treated samples (minimal oxidation observed for TD-3 treated HbS in Table 4 is due to unlabeled Cys93). This work collectively shows that TD-3 has induced changes in oxygen binding affinity of HbS and delayed polymer formation of the deoxy form of Hb *via* protective mechanisms.

Similarly, 5HMF increased the oxygen affinity and delay time of HbS making this molecule an ideal antisickling therapeutic. Specifically, 5HMF acts to inhibit SS cells from sickling by covalently binding to and stabilizing the Hb relaxed state and/or binding to and destabilizing the T state of HbS with a concomitant shift of the allosteric equilibrium to the high affinity HbS. The antisickling activity of 5HMF is primarily due to Schiff-base interactions between the aldehyde moiety and the N-terminal alpha Val1 amine of Hb. Previous X-ray crystallography study supports these observations (Safo et al., 2004). Our results show that there was no change in ferryl heme levels and subsequently no change in irreversible oxidation in βCys93 at low and high levels of H_2_O_2_-treated HbS.

The *in vitro* treatment of HbS with L-glutamine did not show any impact on HbS oxygen affinity nor did it have any effects on Hb’s chemically induced oxidation reactions. The mechanism of action of L-glutamine is believed to be due to its ability to maintain a redox balance within RBCs by increasing internal levels of NAD+. However, the safety and efficacy of L-glutamine therapy in SCD has recently been questioned (Quinn, 2018). Nevertheless, our result on the Hb oxidation showed that L-glutamine did not provide an antioxidant advantage reflected by our spectrophotometric oxidation data and quantitative mass spectrometry results which indicate that glutamine-treated HbS βC93 oxidation was nearly identical to control data when treated with H_2_O_2_. We have also measured effects of these molecules, particularly glutamine on the RBC’s NAD/NADH redox potential. We have carried out measurements on NADH and total NAD in RBCs treated with and without glutamine to assess the change in RBCs redox potential. Treatment of antisickling agents (including glutamine) did not result in any measurable difference in the NADH/total NAD ratio between treated/untreated SCD RBC control (unpublished data). It is known that there is an ongoing debate and many unanswered questions regarding glutamine’s efficacy, safety, and its role in current therapy (Quinn, 2018). We therefore feel that targeting Hb’s own oxidative pathways (specifically βCys93) are more predictive of the overall status of the RBC’s redox state.

In summary, here we show that all four antisickling agents provide some level of antisickling activity (see Table 5). In particular, those antisickling drugs that directly modify βCys93 effectively protect this “hotspot” amino acid from irreversible oxidation by a strong oxidant, such as ferryl heme and simultaneously prolong the delay time prior to the formation of the polymer. Photometric and mass spectrometric studies showed a strong relationship between the levels of ferryl heme and oxidative changes in the Hb molecule. Shielding of βCys93 by these reagents likely prevented the escape of the ferryl radical through this amino acid (Kassa et al., 2015), lessening therefore consequent oxidative damages to the protein. Hence, our results suggest that agents targeting βCys93 of HbS can potentially provide both antisickling and antioxidant therapeutic advantages for patients with SCD.

**Table 5:** Summary of antisickling and antioxidant effects of agents under oxidative stress [2.5 H_2_O_2_/Hb (Heme)].

Agents	O_2_ affinity	Autoxidation rate	Ferryl formation (% Reduction)	Delay time (s)	Cys93 modification
TD-3	Increased	No effect	Reduced (22%)	315	**++**
5-HMF	Increased	No effect	No effect	158	--
HU	Increased	No effect	Reduced (38%)	170	**++**
L-Glutamine	No Effect	No effect	No effect	158	--

*Modified = **++**; not modified = --*.

## Data Availability

The raw data supporting the conclusions of this manuscript will be made available by the authors, without undue reservation, to any qualified researcher.

## Author Contributions

TK and AA designed the work. TK, MS, and FW performed the experiments and data analysis. TK and AA contributed to conception and design. TK, MS, and AA wrote and edited the manuscript.

### Conflict of Interest Statement

The authors declare that the research was conducted in the absence of any commercial or financial relationships that could be construed as a potential conflict of interest.

## Abbreviations

Ferrous Hb: Heme groups contain positively charged iron (Fe^2+^) which can reversibly bind to oxygen molecules
metHb: Ferric (met) hemoglobin, the iron is in the (Fe^3+^)
ferryl Hb: The iron is in the (Fe^4+^)
H_2_O_2_: Hydrogen peroxide
sulfHb: Sulfhemoglobin
AA: Normal RBCs
SS: Homozygous sickle RBCs
HbS: Sickle hemoglobin
ODC: Oxygen dissociation curve
SCD: Sickle cell disease.
